# Editorial: Integrated genomic and systems approaches to unravel host-virus interactions in viral infectious diseases

**DOI:** 10.3389/fcimb.2026.1919905

**Published:** 2026-07-16

**Authors:** Samuel Pushparaj, Megan Justice, Raja Angamuthu, Esteban A. Hernandez-Vargas

**Affiliations:** 1 Oklahoma State University, Stillwater, OH, United States; 2 Tamil Nadu Veterinary and Animal Sciences University, Chennai, India; 3 University of Idaho, Moscow, ID, United States

**Keywords:** genomics, host-virus interactions, innate immunity, interferon signaling, metabolomics, multi-omics, proteomics, systems biology

## Introduction

Viral infectious diseases continue to pose major threats to global public health. Understanding the molecular dialogue between viruses and their hosts is fundamental to developing timely diagnostics, effective treatments, and prevention strategies. Advances in high-throughput sequencing, proteomics, metabolomics, and computational biology have opened unprecedented windows into these interactions. This Research Topic brings together seven original research articles that harness multi-omics and systems-level approaches to illuminate how diverse viral pathogens (spanning herpesviruses, filoviruses, flaviviruses, coronaviruses, and African swine fever virus) engage, subvert, and are countered by host immune and cellular responses. A unifying theme is the pivotal and context-dependent role of the type I interferon (IFN-I) system, alongside the integration of multi-omics data for biomarker and therapeutic target discovery.

## Neurotropic viruses and CNS pathology

Two contributions examine viruses targeting the central nervous system (CNS). A clinical and proteomic analysis of cerebrospinal fluid (CSF) from 69 patients with varicella-zoster virus (VZV) CNS infection documents the predominant manifestations of headache, fever, and motor/sensory disturbances. CSF proteomics revealed markedly elevated protein abundance relative to controls, with upregulated proteins enriched in IFN-1 signaling, pyroptosis, and blood-brain barrier (BBB) disruption pathways. Complement system overactivation in severe encephalitis cases emerged as a candidate marker of disease severity and a potential therapeutic target, underscoring the value of CSF proteomics for identifying actionable molecular signatures.

A multi-system transcriptomic study of Zika virus (ZIKV) integrates data from human brain organoids, monocyte-derived dendritic cells, and PBMCs from infected patients to map interferon-stimulated gene (ISG) regulation across infection stages. IFN-β treatment potently activated ISGs including IRF7 and IFITM3 in brain organoids, while single-cell transcriptomics revealed cellular heterogeneity in ISG responses. Cross-system analysis identified 22 shared ISGs, and LASSO regression nominated 11 diagnostic biomarker candidates. Functional siRNA knockdown confirmed that IFI35, IFI44, and OAS3 restrict ZIKV replication, bridging transcriptomic discovery to validated antiviral mechanisms.

## Systemic infections: Ebola and dengue

A machine learning and mathematical modeling study of Ebola virus disease (EVD) resolves a longstanding controversy over the role of IFN-I in disease outcome. Analysis of patient data from the 2014–2016 West Africa epidemic reveals that IFN-I responses precede symptom onset in survivors, whereas non-survivors mount equivalent responses 3–4 days later. This delay causes IFN-I and IL-12 signals to overlap in non-survivors, disrupting the sequential cytokine cues required for effective T cell activation. The findings elevate temporal immune dynamics as a critical yet underappreciated determinant of host protection versus pathology.

An integrated metabolomics-transcriptomics study of dengue virus (DENV) infection profiles plasma from 41 patients and 23 healthy controls using LC-MS and GC-MS, identifying 197 differentially abundant metabolites. Top upregulated species including trans-cinnamic acid and L-acetylcarnitine are proposed as diagnostic biomarkers by ROC analysis. KEGG and GSEA analyses highlighted disruption of ATP-binding cassette (ABC) transporters as a key metabolic perturbation, corroborated by transcriptomic integration from GEO datasets identifying ABCC5, ABCB1, and ABCG5 as hub targets, opening a new avenue for understanding DENV-induced cellular pathology.

## Coronaviruses: SARS-CoV-2 and animal pathogens

A longitudinal study of 157 COVID-19 patients examines expression of TLR7, TYK2, and OAS1 across disease severity groups. Low TLR7 expression robustly correlates with severity and viremia, while OAS1 persists in severe cases with high viral loads even as TLR7 declines during recovery. The interdependence of these innate immune genes highlights the coordinated yet severity-stratified nature of early antiviral responses to SARS-CoV-2 and has implications for prognostic stratification.

A complementary study examines how transcription factors EB (TFEB) and E3 (TFE3), master regulators of lysosomal biogenesis, modulate viral replication, autophagy, and apoptosis across three coronavirus genera: gamma coronavirus IBV, beta coronavirus HCoV-OC43, and alpha coronavirus PEDV. Infection upregulates TFEB/TFE3 and induces a lysosomal stress response. TFE3 knockdown reveals its predominant role in controlling replication and cell death, with synergistic TFEB/TFE3 effects in IBV- and HCoV-OC43-infected cells. Brefeldin A experiments further establish that HCoV-OC43 egress occurs via the lysosomal pathway, identifying TFEB/TFE3-governed lysosomal biology as a conserved host vulnerability across coronavirus genera.

## African swine fever virus and cell tropism

A transcriptomic investigation of African swine fever virus (ASFV) addresses its strict tropism for primary porcine alveolar macrophages (PAMs) versus limited replication in immortalized macrophage lines (iPAMs). Comparative profiling of the SY-1 strain reveals divergent host responses: iPAMs predominantly engage lipid metabolism and oxidative stress, whereas PAMs activate cytokine signaling and broad immune effector programs. Reduced replication in iPAMs thus reflects a fundamentally different cellular environment, not merely receptor availability. Functional validation identifies JPH4 and CYP1A1 as candidate host permissiveness factors, providing a foundation for improved *in vitro* models and antiviral strategies—critical given the absence of licensed ASF vaccines.

## Conclusion

Together, these seven articles demonstrate the power of integrated multi-omics and systems approaches to decode host-virus interactions. Cross-cutting themes include the centrality and context-dependence of IFN-I signaling, the importance of temporal immune dynamics, the utility of multi-omics integration for biomarker discovery, and the value of appropriate cell and patient models for mechanistic investigation. We thank all contributing authors and reviewers, and hope this collection advances the development of novel diagnostics and host-targeted therapeutics for viral infectious diseases.

